# *Apolipoprotein E epsilon 4 (APOE‐*ε*4)* genotype is associated with decreased 6‐month verbal memory performance after mild traumatic brain injury

**DOI:** 10.1002/brb3.791

**Published:** 2017-08-09

**Authors:** John K. Yue, Caitlin K. Robinson, John F. Burke, Ethan A. Winkler, Hansen Deng, Maryse C. Cnossen, Hester F. Lingsma, Adam R. Ferguson, Thomas W. McAllister, Jonathan Rosand, Esteban G. Burchard, Marco D. Sorani, Sourabh Sharma, Jessica L. Nielson, Gabriela G. Satris, Jason F. Talbott, Phiroz E. Tarapore, Frederick K. Korley, Kevin K.W. Wang, Esther L. Yuh, Pratik Mukherjee, Ramon Diaz‐Arrastia, Alex B. Valadka, David O. Okonkwo, Geoffrey T. Manley, the TRACK‐TBI Investigators

**Affiliations:** ^1^ Department of Neurological Surgery University of California, San Francisco San Francisco CA USA; ^2^ Brain and Spinal Injury Center San Francisco General Hospital San Francisco CA USA; ^3^ Department of Public Health Erasmus Medical Center Rotterdam The Netherlands; ^4^ Department of Psychiatry Indiana University School of Medicine Indianapolis IN USA; ^5^ Program in Medical and Population Genetics The Broad Institute at MIT and Harvard Cambridge MA USA; ^6^ Department of Neurology Harvard Medical School Boston MA USA; ^7^ Department of Bioengineering and Therapeutic Sciences University of California, San Francisco San Francisco CA USA; ^8^ Stritch School of Medicine at Loyola University Maywood IL USA; ^9^ Department of Radiology University of California, San Francisco San Francisco CA USA; ^10^ Department of Emergency Medicine University of Michigan at Ann Arbor Ann Arbor MI USA; ^11^ Departments of Psychiatry and Neuroscience University of Florida Gainesville FL USA; ^12^ Department of Neurology University of Pennsylvania Philadelphia PA USA; ^13^ Department of Neurological Surgery Virginia Commonwealth University Richmond VA USA; ^14^ Department of Neurological Surgery University of Pittsburgh Medical Center Pittsburgh PA USA

**Keywords:** apolipoprotein E, genetic factors, human studies, outcome measures, traumatic brain injury, verbal memory

## Abstract

**Introduction:**

The apolipoprotein E (*APOE*) ε*4* allele associates with memory impairment in neurodegenerative diseases. Its association with memory after mild traumatic brain injury (mTBI) is unclear.

**Methods:**

mTBI patients (Glasgow Coma Scale score 13–15, no neurosurgical intervention, extracranial Abbreviated Injury Scale score ≤1) aged ≥18 years with *APOE* genotyping results were extracted from the Transforming Research and Clinical Knowledge in Traumatic Brain Injury Pilot (TRACK‐TBI Pilot) study. Cohorts determined by *APOE‐*ε*4(+/−)* were assessed for associations with 6‐month verbal memory, measured by California Verbal Learning Test, Second Edition (CVLT‐II) subscales: Immediate Recall Trials 1–5 (IRT), Short‐Delay Free Recall (SDFR), Short‐Delay Cued Recall (SDCR), Long‐Delay Free Recall (LDFR), and Long‐Delay Cued Recall (LDCR). Multivariable regression controlled for demographic factors, seizure history, loss of consciousness, posttraumatic amnesia, and acute intracranial pathology on computed tomography (CT).

**Results:**

In 114 mTBI patients (*APOE‐*ε*4(−)*=79; *APOE‐*ε*4(+)*=35), *ApoE‐*ε*4(+)* was associated with long‐delay verbal memory deficits (LDFR:* B *=* *−1.17 points, 95% CI [−2.33, −0.01], *p *=* *.049; LDCR:* B *=* *−1.58 [−2.63, −0.52], *p *=* *.004), and a marginal decrease on SDCR (*B *=* *−1.02 [−2.05, 0.00], *p *=* *.050). CT pathology was the strongest predictor of decreased verbal memory (IRT:* B *=* *−8.49, SDFR:* B *=* *−2.50, SDCR:* B = *−1.85, LDFR:* B = *−2.61, LDCR:* B = *−2.60; *p *<* *.001). Seizure history was associated with decreased short‐term memory (SDFR:* B = *−1.32, *p *=* *.037; SDCR:* B = *−1.44, *p *=* *.038).

**Conclusion:**

The *APOE‐*ε*4* allele may confer an increased risk of impairment of 6‐month verbal memory for patients suffering mTBI, with implications for heightened surveillance and targeted therapies. Acute intracranial pathology remains the driver of decreased verbal memory performance at 6 months after mTBI.

## 1 INTRODUCTION

Mild traumatic brain injury (mTBI) is a major cause of cognitive impairment, which may be modulated in part by genetic susceptibility. mTBIs constitute 70%–90% of all traumatic brain injuries (TBI) (Cassidy et al., 2004); it is estimated that 20%–25% of patients experience persistent symptoms and/or cognitive and neuropsychiatric deficits at 6–12 months postinjury (Arciniegas, Anderson, Topkoff, & McAllister, 2005). A major health system challenge is that individuals with similar injuries will often manifest different symptoms, follow divergent clinical trajectories, and have varied functional outcomes (Ponsford, Draper, & Schönberger, 2008).

**Figure 1:**
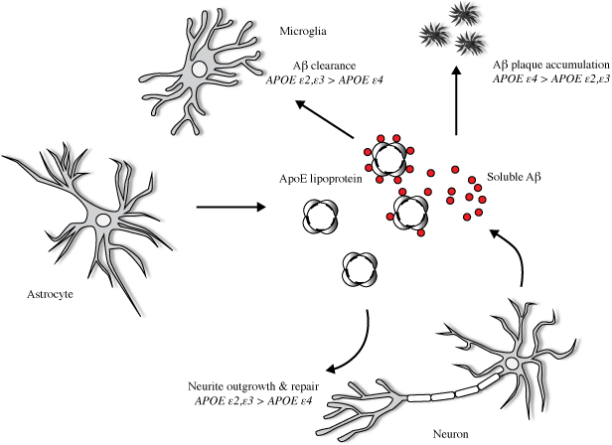
Effects of *APOE* on Aβ metabolism and postinjury repair. Neuronal injury upregulates astrocyte secretion of ApoE, which clears lipid cell debris and assists in cholesterol delivery for synaptogenesis. *APOE* is lipidated to form ApoE lipoprotein, and in the extracellular space binds in an isoform‐dependent pattern (*APOE‐*ε*2*,* ‐*ε*3 >  APOE‐*ε*4*) to soluble beta‐amyloid protein (Aβ), a peptidic neurotoxin. *APOE* genotype determines the capacity for Aβ clearance and parenchymal amyloid plaque accumulation. Evidence suggests that relative to *APOE‐*ε*2* and *‐*ε*3*,*APOE‐*ε*4* is preferentially susceptible to proteolytic degradation, thus reducing the capacity to fulfill postinjury needs for membrane repair and synaptogenesis

Apolipoprotein E (*APOE*) encodes a lipoprotein (ApoE) released primarily by astrocytes after TBI and is known to promote neuronal survival/outgrowth and exert antioxidant effects in the context of oxidative stress and neuroinflammation (Figure 1). ApoE is a key regulator of plasma lipid levels produced in abundance in the brain along with ApoE receptors in order to mediate synaptic repair, remodeling, and protection (Blackman, Worley, & Strittmatter, 2005; Ignatius et al., 1986; Nathan et al., 1994). The gene for ApoE is located on chromosome 19 and is highly polymorphic (Friedman et al., 1999). Differences in the tertiary structure and in the charge distribution of the *APOE* isoforms determine the capacity for cholesterol homeostasis through binding to receptors, to other proteins, and through intracellular trafficking pathways and second messengers (Strittmatter & Bova Hill, 2002; Saito et al., 2003). These differences in metabolic regulation may influence the outcome after brain injury. Of its three common alleles (ε*2*, ε*3*, ε*4*), ε*4*—which has reduced antioxidant and biological activity—is a risk factor for neurodegenerative disorders. While *APOE‐*ε*2* and ε*3,* respectively, contain two and one cysteine residues for detoxifying cytotoxic products of lipid peroxidation, *APOE‐*ε*4* expresses two arginine residues and lacks this ability. Hence, *APOE‐*ε*4* is directly associated with mitochondrial toxicity and beta‐amyloid deposition contributing to Alzheimer's Disease (AD) in a dose‐dependent manner (ε*4* carrier, 5‐fold; ε*4*/ε*4*: 20‐fold) (Blennow, Mattsson, Schöll, Hansson, & Zetterberg, 2015; Hauser & Ryan, 2013).

The *APOE‐*ε*4* allele has been associated with unfavorable outcomes following TBI, including acute clinical deterioration, increased hemorrhage, longer hospital stays, and impaired functional recovery (Chiang, Chang, & Hu, 2003; Jiang et al., 2007; Alexander et al., 2007). This could be due to increased expression of *APOE* in astrocytes following ischemic brain injury and neuronal degeneration (Friedman et al., 1999), furthered by the association between *APOE‐*ε*4* and increased oxidative stress (Jiang et al., 2006). In a landmark study of 984 patients by Teasdale et al. in 2005, the *APOE‐*ε*4* allele was associated with decreased functional recovery after controlling for Glasgow Coma Scale (GCS) motor score, pupillary reactivity, and intracranial computed tomography (CT) pathology; ε*4* carriers under age 45 years were conferred up to 25 additional years of aging after suffering TBI (Teasdale, Murray, & Nicoll, 2005). In moderate and severe TBI patients assessed at least 6 months postinjury, Ariza et al. (2006) found that ε*4*‐carriers performed notably worse on tests of frontal lobe function and long‐term memory. In a large Australian cohort stratified by age, the *APOE‐*ε*4* allele was associated with poorer baseline episodic memory and slower reaction time in younger adults with a history of childhood TBI (Eramudugolla et al., 2014). Similarly, ε*4* carriers may be at more risk for cognitive impairment and chronic traumatic encephalopathy (CTE) (Liberman, Stewart, Wesnes, & Troncoso, 2002; Müller et al., 2009; Lenihan & Jordan, 2015; ). Conversely in children, Moran et al. report that the *APOE‐*ε*4* allele has little impact on neurocognitive measures following TBI, although ε*4*‐carriers were associated with a more negative early response to injury (Moran et al., 2009), which mirrors the adult literature (Friedman et al., 1999; Jiang et al., 2006). Interestingly, pediatric ε*4* carriers sustaining moderate to severe TBI showed poorer adaptive functioning in the context of positive parenting, whereas non‐ε*4‐*carriers displayed worse detriments in the context of less optimal parenting (Treble‐Barna et al., 2016). These studies further highlight the pleiotropic association between *APOE‐*ε*4* and neurobehavioral outcomes after TBI, and underscore the need to elucidate its relationship with outcome subdomains after different types of TBI.

To date, mild‐to‐moderate TBI studies in the literature have reported mixed associations for verbal memory outcome by *APOE‐*ε*4* status (Chamelian, Reis, & Feinstein, 2004; Han et al., 2007; Padgett, Summers, Vickers, McCormack, & Skilbeck, 2016). Interestingly, Han and colleagues reported improved neuropsychological outcome at 1 month was associated with *APOE‐*ε*4* in the military population (Han et al., 2007), whereas Crawford et al. (2002) reported memory and verbal impairment in military personnel with *APOE‐*ε*4* within 6 months of injury. Using an extensive neuropsychological battery at 3 and 6 weeks postinjury, Liberman et al. (2002) reported in adults with mild and moderate TBI that patients with the *APOE‐*ε*4* allele had lower mean scores for nearly all neuropsychological tests and needed more time to complete tasks, although none of the differences were significant. The authors suggested that *APOE* genotype may specifically influence the severity of the acute injury along with delaying recovery. In concussed athletes tested within 6 months of injury, carriers of the ε*4* allele demonstrated greater neurocognitive variability than athletes without an ε*4* allele (Merritt, Rabinowitz, & Arnett, 2017). However, an Indian cohort of mild‐to‐moderate TBI patients at 6 months showed no clear differences in neuropsychological test performance by *APOE‐*ε*4* status (Pruthi et al., 2010). Reports of association between ε*4* carriers and postconcussional symptoms months to years postinjury also exist (Sundström et al., 2007; Merritt & Arnett, 2016; Pan et al., 2016). Population characteristics and TBI severity may contribute to the discrepancies in the current literature. Hence, the association of *APOE‐*ε*4* status and subdomains of verbal memory performance following isolated mTBI warrant further study.

For the current analysis, we utilized data from the prospective multicenter Transforming Research and Clinical Knowledge in Traumatic Brain Injury Pilot (TRACK‐TBI Pilot) study (Yue et al., 2013) to explore associations between *APOE* and components of 6‐month verbal memory postinjury. We demonstrate that the *APOE‐*ε*4* carrier status is specifically associated with decreased performance on long‐delay recall trials, without effects on immediate recall or short‐delay recall trials.

## 2 METHODS

### 2.1 Study design

The TRACK‐TBI Pilot study is a multicenter prospective observational study conducted at three Level I Trauma Centers in the U.S.—San Francisco General Hospital (SFGH), University of Pittsburgh Medical Center (UPMC), and University Medical Center Brackenridge (UMCB) in Austin, Texas—using the National Institutes of Health (NIH) National Institute of Neurological Disorders and Stroke (NINDS) TBI Common Data Elements (CDEs) (Yue et al., 2013; Maas et al., 2010; Duhaime et al., 2010; Manley et al., 2010; Wilde et al., 2010). Inclusion criteria for TRACK‐TBI Pilot were external force trauma to the head presenting to Level I trauma center, and clinically indicated head CT scan within 24 hr of injury. Exclusion criteria were pregnancy, comorbid life‐threatening disease, incarceration, psychiatric hold, and non‐English speaking due to limitations in participation with outcome assessments.

As the goal of the current analysis was to investigate the association between the *APOE‐*ε*4* allele and 6‐month verbal memory outcome after mTBI, uncomplicated by massive intracranial trauma [e.g., CT evidence of mixed density lesions >25 mm, midline shift >5 mm, or evidence of cisternal compression (Marshall et al., 1992)] or polytrauma, patients were included if ≥18 years of age, with GCS score of 13–15 at ED admission per current definition of mTBI (Teasdale & Jennett, 1974; ), Marshall CT Score 1–2 (Marshall et al., 1992), no acute neurosurgical intervention, and no extracranial injuries greater than “mild” (Abbreviated Injury Scale [AIS] score >1 in any extracranial body region). To preserve the integrity of cognitive outcomes analysis, patients with a baseline history or diagnosis of central nervous system (CNS) tumor, cerebrovascular accident (CVA), schizophrenia, bipolar disorder, learning disability, and/or developmental delay were excluded from analysis.

Eligible patients were enrolled through convenience sampling at all three sites. Procedures followed were in accordance with the ethical standards of the responsible committee on human experimentation and with the Helsinki Declaration of 1975, as revised in 2008. Institutional Review Board (IRB) approval was obtained at all participating sites. Informed consent was obtained for all patients prior to enrollment in the study. For patients unable to provide consent due to their injury, consent was obtained from their legally authorized representative (LAR). Patients were then re‐consented, if cognitively able, at later inpatient and/or outpatient follow‐up assessments for continued participation in the study.

### 2.2 Biospecimen acquisition and genotyping

Specimen acquisition was performed as previously described (Yue et al., 2013). In brief, blood samples were collected via peripheral venipuncture or existing peripheral venous or arterial indwelling catheters within 24 hr of injury for DNA genotyping. Samples were collected in BD Vacutainer K_2_‐EDTA vacutainer tubes, then aliquoted and frozen in cryotubes at −80°C within 1 hr of collection in accordance with recommendations from the NIH NINDS CDE Biomarkers Working Group (Manley et al., 2010). DNA was extracted from isolated leukocytes using the Wizard^®^ Genomic DNA Purification Kit as described by the manufacturer (Promega, Madison, WI). *APOE* (rs7412; rs429358) polymorphisms were genotyped using the TaqMan^®^SNP Genotyping Assay (Applied Biosystems, Carlsbad, CA; rs7412 Assay ID# C____904973_10; rs429358 Assay ID# C___3084793_20). *APOE* alleles were determined as ε*2* (rs7412(T)/rs429358(T)), ε*3* (rs7412(C)/rs429358(T), or ε*4* (rs7412(C)/rs429358(C)); no ε*1* cases existed in the dataset. For the purposes of evaluating a potential deleterious effect of the *APOE‐*ε*4* allele, patients with ε*2/*ε*2*, ε*2/*ε*3*, and ε*2/*ε*3* genotypes were grouped as *APOE‐*ε*4(−)*, and ε*3/*ε*4* or ε*4/*ε*4* genotypes were grouped as *APOE‐*ε*4(+)*, similar to previous studies (Teasdale et al., 2005; Sundström et al., 2007).

### 2.3 Outcome measures

The California Verbal Learning Test, Second Edition (CVLT‐II) is a verbal learning and memory task with five learning trials, an interference trial, an immediate recall trial performed with and without cues, and a post‐20 min recall trial performed with and without cues. The CVLT‐II was substituted for the Rey Auditory Verbal Learning Test (RAVLT) listed in the NIH NINDS outcome CDEs due to relevant revisions of the Second Edition and higher consistency on between‐norm sets (Stallings, Boake, & Sherer, 1995). The CVLT‐II has been used in over 200 published clinical and experimental research studies as a measure of episodic verbal learning and memory (Woods, Delis, Scott, Kramer, & Holdnack, 2006). It has also been found to be one of the most sensitive tests for detecting residual brain damage in patients with brain trauma, able to differentiate between outcomes following mild versus moderate‐severe TBI (Davis, 2016; Delis, Kramer, Kaplan, & Ober, 2000). Researchers have found that the CVLT can also accurately predict functional outcomes at the time of testing, including level of current disability, type of current supervision, and return to work status (Hanks, Jackson, & Crisanti, 2016).

The CVLT‐II subscales allow for differentiation between encoding, consolidation, and retrieval deficits (Vanderploeg, Crowell, & Curtiss, 2001). Lower scores on the CVLT‐II Short‐Delay Free Recall (SDFR) indicate retroactive interference, while lower scores on the CVLT‐II Long‐Delay Free Recall (LDFR) score indicate the occurrence of rapid forgetting. Free recall trials reflect the degree to which words on the first list are recalled without assistance. Cued recall trials assist the examinee in two ways: (1) by informing them of the categorical structure of the list; and (2) requiring them to use semantic clustering in recalling the target words. Patients with mild cognitive impairment (MCI) will often demonstrate aberrant responses on cued recall trials. Patients with more severe deficits will typically respond with deficits on both free and cued recall trials.

### 2.4 Statistical analysis

Descriptive statistics are presented as means and standard deviations (SD) for continuous variables and as proportions for categorical variables. Group differences in demographics and injury characteristics across *APOE‐*ε*4* genotypes were assessed by Pearson's chi‐squared test (*X* ^*2*^) for categorical variables, and analysis of variance (ANOVA) for continuous variables. Fisher's Exact Test was used to assess for differences in categorical variables with individual cell counts ≤5. Multivariable linear regression was performed to assess the association between *APOE‐*ε*4* genotype and each of the five CVLT‐II outcome measures. Demographic and injury variables were selected based on recommendations and cited predictive value in large studies and systematic reviews in mTBI to include age in years (scalar), education in years (scalar), sex (male vs. female), race (dichotomized to Caucasian vs. other due to small samples of other races), loss of consciousness (LOC; no vs. yes/unknown), posttraumatic amnesia (PTA; no vs. yes/unknown), and presence of intracranial pathology on initial head computed tomography (CT) scan (Jacobs et al., 2010; Kashluba, Hanks, Casey, & Millis, 2008; Van der Naalt, 2001; Carroll et al., 2004). A history of seizures has been correlated with decreased verbal memory performance in the neurology literature and hence prior medical history (PMH) of seizures was included to serve as a surrogate for baseline cognitive burden (Blake, Wroe, Breen, & McCarthy, 2000; Schefft et al., 2008; Berl et al., 2005). To prevent overfitting, we adhered to the rule of 10 for multiple regression analysis (predictors=9; *n* = 114) (Howell, 2013; Green, 1991; ). To account for attributable variance (*R* ^*2*^), a two‐tiered approach was employed for each multivariable regression model. First, demographic and injury history predictors were entered onto the model for each CVLT‐II outcome measure and *R* ^*2*^ was recorded (Table S1); then, *APOE‐*ε*4* status was entered onto the model, and the new *R* ^*2*^ as well as the *ΔR* ^*2*^ from the model without *APOE‐*ε*4* status was calculated and reported. The multivariable regression mean differences (*B*) and their associated 95% confidence intervals (CI) are reported for each predictor in the regression analyses. When appropriate, the regression means and standard errors (SE) are reported for *APOE‐*ε*4(+)* and *APOE‐*ε*4(−)* cohorts. Significance was assessed at α = 0.05. All analyses were performed using Statistical Package for the Social Sciences (SPSS) version 22 (IBM Corporation, Chicago, IL, USA).

## 3 RESULTS

### 3.1 Demographic and injury characteristics

In total, 114 patients were included in the current analysis (Table 1). The majority were male (67%) and of Caucasian race (73%). Mean age was 42.8 ± 16.2 years, and education level was 14.2 ± 2.9 years. Mechanisms of injury included fall (46%), motor vehicle accident (21%), cyclist/pedestrian struck by vehicle (14%), assault (14%), and struck by/against object (4%). Approximately, 12% of patients reported PMH of seizures, while 23% reported absence of LOC and 40% reported absence of PTA. ED GCS deficit (<15) occurred in 20% of patients, and 25% of patients showed evidence of acute intracranial pathology on initial head CT. *APOE* genotypes were distributed as follows: ε*2/*ε*3 n* = 12 (11%), ε*3/*ε*3 n* = 67 (59%), ε*3/*ε*4 n* = 29 (25%), ε*2/*ε*4 n* = 3 (3%), and ε*4/*ε*4 n* = 3 (3%). Breakdown by ethnicities were: Caucasian: ε2/ε2 = 11, ε3/ε3 = 47, ε3/ε4 = 22, ε2/ε4 = 1, ε4/ε4 = 2; African‐American/African: ε2/ε4 = 2, ε3/ε3 = 7, ε3/ε4 = 6, ε4/ε4 = 1; other races: ε2/ε3 = 1, ε3/ε3 = 13, ε3/ε4 = 1. Overall, 31% of patients were *APOE‐*ε*4(+)*. *APOE* distribution conformed to Hardy–Weinberg equilibrium (allelic frequencies: ε*2 *=* *0.0658, ε*3 *=* *0.7675, ε*4 *=* *0.1667; χ^2^ =0.554, df=5, *p *=* *.990).

**Table 1:** Demographic and clinical information of included patients with mild traumatic brain injury

Variable	Overall (*N* = 114)	*APOE* ε*4(−)* (*N* = 79)	*APOE* ε*4(+)* (*N* = 35)	Sig. (*p*)
Age (years)				
Mean ± *SD*	42.8 ± 16.2	39.7 ± 16.5	49.6 ± 13.6	.002
Gender				
Male	76 (67%)	49 (62%)	27 (77%)	.114
Female	38 (33%)	30 (38%)	8 (23%)	
Race				
Caucasian	83 (73%)	58 (70%)	25 (30%)	.012
African‐American/African	16 (14%)	7 (44%)	9 (56%)	
Other races	15 (13%)	14 (93%)	1 (7%)	
PMH Seizures				
No	100 (88%)	70 (89%)	30 (86%)	.664
Yes	14 (12%)	9 (11%)	5 (14%)	
Education (years)				
Mean ± *SD*	14.2 ± 2.9	14.3 ± 3.1	13.7 ± 2.1	.280
Mechanism of injury				
Motor vehicle accident	24 (21%)	17 (22%)	7 (20%)	.910
Cyclist/pedestrian hit	16 (14%)	11 (14%)	5 (14%)	
Fall	53 (47%)	38 (48%)	15 (43%)	
Assault	16 (14%)	10 (13%)	6 (17%)	
Struck by/against object	5 (4%)	3 (4%)	2 (6%)	
Loss of consciousness				
No	26 (23%)	19 (24%)	7 (20%)	.634
Yes/unknown	88 (77%)	60 (76%)	28 (80%)	
Posttraumatic amnesia				
No	46 (40%)	30 (38%)	16 (46%)	.437
Yes/unknown	68 (60%)	49 (62%)	19 (54%)	
ED arrival GCS				
13–14	23 (20%)	16 (20%)	7 (20%)	.975
15	91 (80%)	63 (80%)	28 (80%)	
CT pathology				
Absent	85 (75%)	58 (73%)	27 (77%)	.674
Present	29 (25%)	21 (27%)	8 (23%)	

Race distributions are reported as row percentages. All other distributions reported as column percentages. The race subgroup “Other races” was combined due to individual small sample sizes of Asian [*N* = 6; ε*4(−) *= 5, ε*4(+) = *1], American Indian/Alaskan Native [*N* = 1; ε*4(−) *= 1], Hawaiian/Pacific Islander [*N* = 2; ε*4(−) *= 2], and more than one race [*N* = 6; ε*4(−) *= 6]. ED Arrival GCS was combined due to *N* = 1 for GCS 13 in both *APOE* groups.

*APOE*, apolipoprotein E; CI, confidence interval; ED, emergency department; GCS, Glasgow Coma Scale; PMH, prior medical history; *SD*, standard deviation.

Demographic and injury characteristics by *APOE‐*ε*4* carrier status are shown in Table 1. Notably, *ApoE‐*ε*4(+)* patients were older (49.6 vs. 39.7 years, *p *=* *.002). *APOE‐*ε*4* carrier status differed across races, with African‐American/African patients showing the highest incidence, followed by Caucasians (*p *=* *.012); the higher prevalence of the ε*4* allele in African‐American/African patients, and of the ε*3* allele in Caucasians, is consistent with prior epidemiological reports (Farrer et al., 1997; Kern et al., 2015). No other demographic or injury history differences were observed by *APOE‐*ε*4* carrier status.

### 3.2 *APOE‐*ε*4*(+) is not associated with immediate recall or short‐delay recall at 6 months postinjury

For IRT, no significant differences were observed by *APOE‐*ε*4* status (*p *=* *.190). Increasing age was associated with decreasing recall (per‐year *B = *−0.28, 95% CI [−0.40, −0.16]; *p *<* *.001). Increasing education was associated with improved recall (per‐year *B = *0.89 [0.26, 1.53]; *p *=* *.006). Caucasian race was associated with a 5.42‐point increase ([1.33, 9.50], *p *=* *.010) compared to other races. PTA+ patients were associated with a 5.15‐point decrease ([−9.01, −1.19], *p *=* *.011). Patients with intracranial CT pathology showed a mean decrease of 8.49‐points ([−12.64, −4.34], *p *<* *.001) (Table 2). The *ΔR* ^*2*^ from adding *APOE‐*ε*4* status was +0.010.

**Table 2:** Multivariable regression of *APOE‐*ε*4* status and 6‐month verbal memory subscales

Variable	*B* [95% CI]	Sig. (*p*)
Immediate Recall Trials 1–5 (*R* ^2^ = 0.405; Δ*R* ^2^ = +0.010)		
*APOE‐*ε*4(+)*	−2.69 [−6.72, 1.36]	.190
Age (per‐year)	−0.28 [−0.40, −0.16]	<.001
Education (per‐year)	0.89 [0.26, 1.53]	.006
Sex (male)	−0.28 [−4.15, 3.59]	.886
Race (Caucasian)	5.42 [1.33, 9.50]	.010
PMH Seizures (yes)	−4.13 [−9.53, 1.26]	.132
LOC (yes/unknown)	1.07 [−3.48, 5.62]	.641
PTA (yes/unknown)	−5.15 [−9.10, −1.19]	.011
CT Pathology (yes)	−8.49 [−12.64, −4.34]	<.001
Short‐Delay Free Recall (*R* ^2^ = 0.356; Δ*R* ^2^ = +0.012)		
*APOE‐*ε*4(+)*	−0.99 [−2.27, 0.29]	.129
Age (per‐year)	−0.08 [−0.12, −0.04]	<.001
Education (per‐year)	0.23 [0.03, 0.44]	.023
Sex (male)	0.01 [−1.21, 1.24]	.985
Race (Caucasian)	1.38 [0.08, 2.68]	.037
PMH Seizures (yes)	−1.32 [−3.03, 0.39]	.129
LOC (yes/unknown)	0.55 [−0.89, 1.99]	.451
PTA (yes/unknown)	−0.99 [−2.24, 0.29]	.122
CT Pathology (yes)	−2.50 [−3.81, −1.18]	<.001
Short‐Delay Cued Recall (*R* ^2^ = 0.380; Δ*R* ^2^ = +0.023)		
*APOE‐*ε*4(+)*	−1.02 [−2.05, 0.00]	.050
Age (per‐year)	−0.07 [−0.10, −0.04]	<.001
Education (per‐year)	0.18 [0.02, 0.34]	.029
Sex (male)	0.50 [−0.48, 1.48]	.310
Race (Caucasian)	1.15 [0.12, 2.19]	.029
PMH Seizures (yes)	−1.44 [−2.81, −0.08]	.038
LOC (yes/unknown)	0.27 [−0.88, 1.42]	.644
PTA (yes/unknown)	−1.03 [−2.03, −0.03]	.043
CT Pathology (yes)	−1.85 [−2.90, −0.85]	<.001
Long‐Delay Free Recall (*R* ^2^ = 0.415; Δ*R* ^2^ = +0.022)		
*APOE‐*ε*4(+)*	−1.17 [−2.33, −0.01]	.049
Age (per‐year)	−0.08 [−0.11, −0.04]	<.001
Education (per‐year)	0.22 [0.03, 0.42]	.010
Sex (male)	0.58 [−0.53, 1.69]	.304
Race (Caucasian)	1.69 [0.58, 2.87]	.005
PMH seizures (yes)	−1.49 [−3.05, 0.06]	.059
LOC (yes/unknown)	0.45 [−0.86, 1.76]	.499
PTA (yes/unknown)	−1.28 [−2.42, −0.14]	.028
CT pathology (yes)	−2.61 [−3.80, −1.42]	<.001
Long‐Delay Cued Recall (*R* ^2^ = 0.408; Δ*R* ^2^ = +0.050)		
*APOE‐*ε*4(+)*	−1.58 [−2.63, −0.52]	.004
Age (per‐year)	−0.06 [−0.09, −0.03]	<.001
Education (per‐year)	0.16 [−0.01, 0.32]	.062
Sex (male)	0.44 [−0.57, 1.45]	.386
Race (Caucasian)	1.14 [0.07, 2.20]	.038
PMH Seizures (yes)	−0.96 [−2.38, 0.45]	.180
LOC (yes/unknown)	0.35 [−0.83, 1.54]	.556
PTA (yes/unknown)	−0.69 [−1.73, 0.34]	.186
CT Pathology (yes)	−2.60 [−3.69, −1.52]	<.001

Tier 2 of the hierarchical multivariable linear regression with the response variables being five verbal memory subscales of the California Verbal Learning Test, Second Edition (CVLT‐II). Mean increase or decrease (*B)* and associated 95% confidence intervals [95% CI] are reported for each predictor.

*APOE*, apolipoprotein E; CT, computed tomography; LOC, loss of consciousness; PMH, prior medical history; PTA, posttraumatic amnesia.

**Figure 2:**
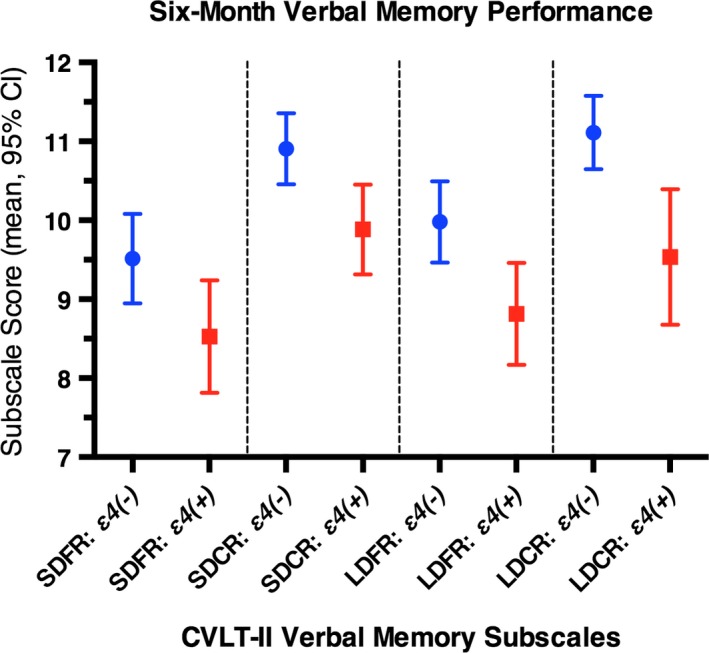
Six‐month verbal memory performance, by *APOE‐*ε*4* carrier status. Performance on four CVLT‐II subscales (SDFR, Short‐Delay Cued Recall (SDCR), Long‐Delay Free Recall (LDFR), LDCR) at 6 months postinjury are shown for 114 mTBI patients, by *APOE‐*ε*4* carrier status. Raw scores for each CVLT‐II subscale are controlled for age, education, sex, race, prior medical history (PMH) seizures, loss of consciousness, posttraumatic amnesia, and acute intracranial pathology on head CT scan. Multivariable regression means and 95% confidence intervals are shown for *APOE‐*ε*4* groups. *APOE‐*ε*4(−)*, blue; *APOE‐*ε*4(+)*, red. *APOE*, apolipoprotein E; CT, computed tomography; CVLT‐II, California Verbal Learning Test, Second Edition; LDCR, Long‐Delay Cued Recall; LDFR, Long‐Delay Free Recall; mTBI, mild traumatic brain injury; PMH, prior medical history; SDCR, Short‐Delay Cued Recall; SDFR, Short‐Delay Free Recall

For SDFR, no significant differences were observed by *APOE‐*ε*4* status (*p *=* *.129) (Figure 2). Age was associated with decreased recall (per‐year *B = *−0.08 [−0.12, −0.04]; *p *<* *.001). Education was associated with improved recall (per‐year *B = *0.23 [0.03, 0.44]; *p *=* *.023). Caucasian race (*B *=* *1.38 [0.08, 2.68], *p *=* *.037) and intracranial CT pathology (*B *=* *−2.50 [−3.81, −1.18], *p *<* *.001) remained predictors. The *ΔR* ^*2*^ from adding *APOE‐*ε*4* status was +0.012.

For Short‐Delay Cued Recall (SDCR), *APOE‐*ε*4(+)* patients showed a marginal decrease in performance (*B *=* *−1.02 [−2.05, 0.00], *p *=* *.050) compared with their *APOE‐*ε*4(−)* counterparts (Figure 2). Age was associated with decreased recall (per‐year *B = *−0.07 [−0.10, −0.04]; *p *<* *.001). Education was associated with improved recall (per‐year *B = *0.18 [0.02, 0.34]; *p *=* *.029). Caucasian race remained protective (*B *=* *1.15 [0.12, 2.19), *p *=* *.029), while CT pathology was deleterious (*B *=* *−1.85 [−2.90, −0.85], *p *<* *.001). Having experienced PTA (*B *=* *−1.03 [−2.03, −0.03], *p *=* *.043) and PMH of seizures (*B *=* *−1.44 [−2.81, −0.08], *p *=* *.038) emerged as predictors. The Δ*R* ^*2*^ from adding *APOE‐*ε*4* status was +0.023.

### 3.3 *APOE‐*ε*4(+)* is associated with impaired long‐delay recall at 6 months postinjury

For LDFR, *APOE‐*ε*4(+)* patients showed a significant decrease in verbal memory function (*B *=* *−1.17 [−2.33, −0.01], *p *=* *.049) compared with *APOE‐*ε*4(−)* (Figure 2). Age was associated with decreased recall (per‐year *B = *−0.08 [−0.11, −0.04]; *p *<* *.001). Education was associated with improved recall (per‐year *B = *0.22 [0.03, 0.42]; *p *=* *.010). Caucasian race remained protective (*B *=* *1.69 [0.58, 1.69], *p *=* *.005), while PTA+ (*B *=* *−1.28 [−2.42, −0.14], *p *=* *.028) and CT pathology remained deleterious (*B *=* *−2.61 [−3.80, −1.42], *p *<* *.001). The *ΔR* ^*2*^ from adding *APOE‐*ε*4* status was +0.022.

For Long‐Delay Cued Recall (LDCR), *APOE‐*ε*4(+)* patients showed a similar decrease in recall ability (*B *=* *−1.58 [−2.63, −0.52], *p *=* *.004) compared with *APOE‐*ε*4(−)* (Figure 2). Age was associated with decreased recall (per‐year *B = *−0.06 [−0.09, −0.03]; *p *<* *.001). Education was associated with marginally improved recall (per‐year *B = *0.16 [−0.01, 0.32]; *p *=* *.062). Caucasian race remained protective (*B *=* *1.14 [0.07, 2.20], *p *=* *.038), while CT pathology (*B *=* *−2.60 [−3.69, −1.52], *p *<* *.001) continued to be deleterious to verbal memory function. The Δ*R* ^*2*^ from adding *APOE‐*ε*4* status was +0.050.

## 4 DISCUSSION

In a prospective study examining the association between *APOE‐*ε*4* and verbal memory deficits after isolated mTBI, we find that *APOE‐*ε*4(+)* is independently associated with impaired long‐delay free and cued recall at 6 months postinjury when compared with *APOE‐*ε*4(−)* patients. We also demonstrate that intracranial CT pathology remains a strong independent driver of decreased 6‐month verbal memory performance after mTBI.

### 4.1 APOE expression, distribution, and function

ApoE is synthesized primarily by astrocytes, with some neuronal expression (Aoki et al., 2003; Orihara & Nakasono, 2002; ] and used for cell membrane repair, growth of neurites, and synaptogenesis. ApoE facilitates neuronal repair processes in deep brain structures including the hippocampus, entorhinal complex, parahippocampal gyrus, and basal ganglia (Crawford et al., 2002), areas essential to memory consolidation processes which are consistently damaged following TBI (Bigler, 1987; Bigler, Anderson, & Blatter, 2002; Graham, Lawrence, Adams, Doyle, & McLellan,^1988^). Studies measuring cerebral blood flow (CBF) demonstrated that *APOE‐*ε*4(+)* patients showed negative associations between verbal memory and decreased CBF in the medial frontal cortex, insula, and basal ganglia (Zlatar et al., 2016). Nathoo et al. noted that *APOE‐*ε*4* carriers show a greater propensity for developing age‐related cognitive impairment, a decrease in the synapse‐neuron ratio, and an increased susceptibility to exogenous neurotoxins and hippocampal atrophy independent of head trauma (Nathoo, Chetry, van Dellen, Connolly, & Naidoo, 2003).

Increasing evidence suggests that ApoE‐ε4 can impede recovery via several mechanisms, including less efficient lipid transport, more beta‐amyloid accumulation, and predisposition to cerebral amyloid angiopathy (Kay et al., 2003; Nicoll, Roberts, & Graham, 1995; Greenberg, Rebeck, Vonsattel, Gomez‐Isla, & Hyman, 1995), increased inflammatory response, and impaired cerebral perfusion (Grocott et al., 2001; Kerr, Kraus, Marion, & Kamboh, 1999), as well as compromised blood–brain barrier and marked cerebral edema (Methia et al., 2001; Lynch et al., 2002). Previous work from the veteran population have shown that *APOE‐*ε*4(+)* patients experienced more difficulty with memory compared to those who were *APOE‐*ε*4(−)* following equally severe TBI, as measured on the CVLT‐II verbal memory and fluency measures at 6 months (Crawford et al., 2002). Similarly, Anderson et al. (2009)showed poorer verbal intellectual and verbal memory skills in those who were *APOE‐*ε*4(+)*, although the differences were not statistically significant after adjusting for multiple comparisons. Of note, ApoE‐ε4 has been associated with verbal memory deficits in mTBI but not with the other aspects of executive function. Significant hippocampal atrophy and temporal horn enlargement have a tendency to occur after TBI, and between 2–7 months postinjury, temporal horn volumes correlated with measured intelligence quotients, while hippocampal volume correlated with verbal memory function (Bigler et al., 2002). Findings in APOE‐knockout mice after TBI demonstrated marked learning deficits associated with neuronal death specifically in the hippocampus. Thus, ApoE is proposed to play a key role in the neuronal repair of the hippocampus and the surrounding regions through isoform‐dependent clearance of beta‐amyloid depositions after trauma (Fagan & Holtzman, 2000).

### 4.2 *APOE‐*ε*4(+)* associates with impaired long‐term verbal memory following mTBI

The CVLT‐II tests encoding, consolidation, and retrieval. The long‐delay recall subtests assess forgetting rates over longer intervals and provide a score of retention in the absence of retroactive interference (Delis et al., 2000). Encoding measures the relative rate of learning. Consolidation is a postencoding process that involves maintenance, elaboration, and storage of new information in long‐term memory (LTM). Evidence in the moderate and severe TBI literature has yielded mixed results—Vanderploeg et al. (2001) report no difference in encoding did not differ between TBI and controls on SDFR and SDCR, while Noe, Ferri, Colomer, Moliner, and Chirivella (2010) found *APOE* genotype to be associated with the trajectory of cognitive recovery but not in memory rehabilitation. In concussed athletes, *APOE‐*ε*4(+)* patients demonstrated greater neurocognitive variability compared to noncarriers (Merritt et al., 2017), and in mild‐to‐moderate TBI patients, Lieberman reported nonsignificant decreases in performance across a range of neuropsychological tests for *APOE‐*ε*4(+)* patients (Liberman et al., 2002). Similarly, in a small study of 34 patients following mTBI, *APOE‐*ε*4(+)* was associated with decreased within‐person postinjury performance on three of nine neuropsychological tests (Sundstrom et al., 2004). Interestingly and importantly we find that, in mTBI patients, subtests of encoding (IRT, SDFR, SDCR) are unaffected or marginally affected by *APOE‐*ε*4(+)* status, while subtests of consolidation are worsened by *APOE‐*ε*4(+)* status. Hence, we extend the findings that information not properly consolidated will rapidly decay from LTM, thus becoming poorly recognized on LDFR and LDCR (Vanderploeg et al., 2001). While the *R* ^*2*^ values for the regression models are relatively low, they are reasonable for human studies; the 2.2%–5.0% increase in *R* ^*2*^ from the addition of *APOE‐*ε*4* status for LDFR and LDCR, while small, is in line with expectations in a multifactorial model consisting of other validated predictors for mTBI outcome, and is deserving of attention considering it is from a single gene, but should not be overinterpreted. The value, and equipoise, of evaluating *APOE* as a locus of susceptibility and a target for intervention following mTBI remains to be determined in future prospective studies of larger scale and rigorous study design.

Padgett and colleagues describe in a literature review that the relationship between *APOE* and cognitive function following TBI is complex and requires a nuance‐based approach—which can be partially elucidated through analysis of specific deficits in appropriate TBI subpopulations (e.g., mTBI vs. moderate/severe‐TBI; isolated TBI vs. polytrauma) (Padgett et al., 2016). Following mTBI, episodic verbal memory deficiencies are greatest between complicated mTBI and healthy controls (Tayim, Flashman, Wright, Roth, & McAllister, 2016). Our analysis replicates this phenomenon, as CT pathology was the driver of decreased cognitive performance following mTBI, with greater deleterious associations with consolidation and retrieval rather than encoding. This supports the idea that following ischemia and neuronal damage, the reduced antioxidant and biological activity of the ε*4* allele may exacerbate vascular endothelial injury (Bell et al., 2012; Halliday et al., 2016) and lead to cognitive deficits (Friedman et al., 1999). Similar results have been described in AD patients with left temporal and/or hippocampal damage, where a subtle decline in episodic memory occurs prior to the emergence of full dementia. The presence of the *APOE‐*ε*4* allele increases deposition of beta‐amyloid plaques, and has been shown to independently contribute to the prediction of conversion dementia when measured by tests of episodic memory such as the CVLT‐II (Lange et al., 2002).

### 4.3 *APOE‐*ε*4(+)*, demographics, and outcome

Age and education effects on the CVLT‐II subscales have been previously described (Delis et al., 2000). There is a known association between increasing age and decreasing performance (Delis et al., 2000; Wiens, Tindall, & Crossen, 1994; Paolo, Troster, & Ryan, 1997) and hence the CVLT‐II manual recommends controlling for age when analyzing CVLT‐II performance (Delis et al., 2000). In our sample, age was a statistically significant independent predictor of poorer verbal memory performance across all CVLT‐II subscales, which may be due to a combination of normal cerebral aging processes leading to slower verbal memory processing—a finding consistent with prior reports in mTBI (Jacobs et al., 2010). It should be noted that in our sample, *APOE‐*ε*4(+)* patients were on average 10 years older than ε*4(−)* patients, which may further compound their decreased verbal memory performance of the ε*4*‐carriers. This also constitutes a limitation of the current analysis, and future studies should incorporate ε*4(+)* and ε*4(−)* cohorts matched by age (e.g., *APOE‐*ε*4(+)* individuals vs. *APOE‐*ε*4(−)* “controls”) in the study design. Education years was also an independent predictor of verbal memory outcomes following mTBI in the current analysis. Previous reports regarding education effects on the CVLT‐II are more unclear; Paolo et al. (1997) report that education affects only 14% of CVLT‐II indices, and Wiens et al. (1994) did not find a consistent contribution of education to CVLT‐II. However, education is a known predictor of protracted return to work following mTBI (Stulemeijer, van der Werf, Borm, & Vos, 2007), in part as a proxy for baseline reading and literacy capabilities (Dotson, Kitner‐Triolo, Evans, & Zonderman, 2009; Albert & Teresi, 1999; ), as well as postinjury coping/resilience, socioeconomic status, and social support.

The relationship between race and CVLT‐II has been previously described (Delis et al., 2000; Nathoo et al., 2003). In our study, race was associated with CVLT performance after injury. The reason for this is unclear, and may be related to a variety of factors. The ε*4* allele is more common in individuals of African‐American/African descent compared with Caucasians (Farrer et al.,^1997^; Kern et al., 2015), and evidence suggests that ε*4* allele effects are weaker in the former (Kaup et al., 2015). Caucasian patients are more likely to have better access to healthcare and rehabilitation following TBI (Gasquoine, 2009; ). Further, recent reports show that African‐American and Hispanic patients are less likely to be placed in acute rehabilitation (Shafi et al., 2007). Hence, racial differences in access to care, and the quality of care received, may additionally influence CVLT performance following mTBI. *APOE‐*ε*4* has also been implicated in differential predilections for developing cerebrovascular disease across ethnicities (Gavett, John, Gurnani, Bussell, & Saurman, 2015). These represent important future directions for the study of *APOE* in mTBI.

### 4.4 Limitations

This study has a relatively small population (*n* = 114), which may have impacted the true strength of association between *APOE‐*ε*4(+)* and short‐delay recall trials, as well as limiting the applicability of our study to all mTBI patients. Due to our small sample size, we were unable to control for additional confounders without risk of overfitting the regression model, which further limits the generalizability of our results. The deleterious impacts on verbal memory attributable to *APOE* were also relatively small, especially in context of positive CT pathology. While there is some evidence that *APOE‐*ε*2* may confer a protective effect on cognitive health (Suri, Heise, Trachtenberg, & Mackaey, 2013), we were not able to investigate this aside from *APOE‐*ε*4* effects due to small sample size. While we controlled for race using multivariable analysis, we were unable to assess differences attributable to *APOE‐*ε*4(+)* within individual races. *APOE* effects in the context of disparate patterns of cognitive decline by race (Gu et al., 2015) present an additional confounder that cannot be addressed in detail by this study. Also, as the CVLT‐II was administered in English, patients whose first language is not English may perform with subtle differences. While out of scope of the current analysis, the long‐term associations between *APOE‐*ε*4* and outcomes after mTBI also warrant further investigation; another study in 396 severe TBI patients found no clear differences between ε4‐carriers and noncarriers on neuropsychological test performance at a follow‐up interval of 15–26 years postinjury (Millar, Nicoll, Thornhill, Murray, & Teasdale, 2003), while a separate study reports poorer cognitive functioning in *APOE‐*ε*4(+)* individuals three decades after TBI (Isoniemi, Tenovuo, Portin, Himanen, & Kairisto, 2006; ). Finally, as stated previously, the attributable R2 change to *APOE‐*ε*4* status is relatively small in the context of other validated predictors for mTBI outcome, and the value of *APOE* as a target for therapeutic evaluation following mTBI remains to be determined pending future work toward elucidating molecular mechanisms, and defining the criteria for heightened surveillance and/or intervention.

The TRACK‐TBI Pilot study was also limited by the variables of the NIH NINDS TBI CDEs version 1, which did not include the highest level of granularity for certain periinjury measures, for example, scalar duration in minutes and/or hours of LOC and PTA which may have been useful for estimating relative mTBI severity. Additionally, the NIH NINDS TBI CDEs version 1 recommended follow‐up using neurocognitive measures at the single time point of 6 months, which prevents analysis of verbal memory recovery over time. These issues will be better addressed by the larger sample size and multiple postinjury outcomes assessment time points as part of the ongoing 12‐center Transforming Research and Clinical Knowledge in Traumatic Brain Injury study in the U.S. (TRACK‐TBI; http://tracktbi.ucsf.edu) and 22‐country Collaborative European NeuroTrauma Effectiveness Research in Traumatic Brain Injury (CENTER‐TBI; http://www.center-tbi.eu) study in the European Union (Maas et al., 2015).

## 5 CONCLUSIONS

Presence of the *APOE‐*ε*4* allele is associated with decreased long‐term verbal memory on two subscales of the CVLT‐II independent of demographics, injury history, and intracranial CT pathology, suggesting disruption to consolidation and retrieval processes following mTBI. These results support and extend previous findings between *APOE‐*ε*4* and impaired cognitive function, and preliminarily strengthen the link between long‐term verbal memory deficits after mTBI and the pathophysiology of neurodegenerative disorders. Future prospective studies targeting *APOE‐*ε*4* are needed to validate these findings.

## CONFLICT OF INTEREST

The authors declare no conflicts of interest.

## DATA AND RESEARCH MATERIALS TRANSPARENCY STATEMENT

The data used in this study are stored in the Federal Interagency Traumatic Brain Injury Research (FITBIR) informatics system (https://fitbir.nih.gov/) and are available for access to qualified researchers.

## Appendix 1 TRACK‐TBI Investigators

### 1.1

Shelly R. Cooper, BA (Department of Psychology, Washington University in St. Louis, St. Louis, MO), Kristen Dams‐O'Connor, PhD (Department of Rehabilitation Medicine, Mount Sinai School of Medicine, New York, NY), Celeste Eng, MS (Department of Bioengineering and Therapeutic Sciences, University of California, San Francisco, San Francisco, CA), Wayne A. Gordon, PhD (Department of Rehabilitation Medicine, Mount Sinai School of Medicine, New York, NY), Allison J. Hricik, MS (Department of Neurosurgery, University of Pittsburgh Medical Center, Pittsburgh, PA), Donglei Hu, PhD (Department of Bioengineering and Therapeutic Sciences, University of California, San Francisco, San Francisco, CA), Joel H. Kramer, PsyD (Department of Neurology, University of California, San Francisco, San Francisco, CA), Andrew I. R. Maas, MD, PhD (Department of Neurosurgery, Antwerp University Hospital, Edegem, Belgium), David K. Menon, MD, PhD (Division of Anaesthesia, University of Cambridge, Addenbrooke's Hospital, Cambridge, United Kingdom), Sam S. Oh (Department of Bioengineering and Therapeutic Sciences, University of California, San Francisco, San Francisco, CA), Ava M. Puccio, RN, PhD (Department of Neurological Surgery, University of Pittsburgh, Pittsburgh, PA), David M. Schnyer, PhD (Department of Psychology, University of Texas at Austin, Austin, TX), Mary J. Vassar, RN, MS (Department of Neurological Surgery, University of California, San Francisco, San Francisco, CA).

## References

[brb3791-bib-0001] Albert, S. M. , & Teresi, J. A. (1999). Reading ability, education, and cognitive status assessment among older adults in Harlem, New York City. American Journal of Public Health, 89, 95–97. 9987476 10.2105/ajph.89.1.95 PMC1508491

[brb3791-bib-0002] Alexander, S. , Kerr, M. E. , Kim, Y. , Kamboh, M. I. , Beers, S. R. , & Conley, Y. P. (2007). Apolipoprotein E4 allele presence and functional outcome after severe traumatic brain injury. Journal of Neurotrauma, 24, 790–797. 17518534 10.1089/neu.2006.0133

[brb3791-bib-0003] Anderson, G. D. , Temkin, N. R. , Dikmen, S. S. , Diaz‐Arrastia, R. , Machamer, J. E. , Farhrenbruch, C. , … Sadrzadeh, S. M. (2009). Haptoglobin phenotype and apolipoprotein E polymorphism: Relationship to posttraumatic seizures and neuropsychological functioning after traumatic brain injury. Epilepsy & Behavior, 16, 501–506. 19766540 10.1016/j.yebeh.2009.08.025 PMC2783358

[brb3791-bib-0004] Aoki, K. , Uchihara, T. , Sanjo, N. , Nakamura, A. , Ikeda, K. , Tsuchiya, K. , & Wakayama, Y. (2003). Increased expression of neuronal apolipoprotein E in human brain with cerebral infarction. Stroke, 34, 875–880. 12649507 10.1161/01.STR.0000064320.73388.C6

[brb3791-bib-0005] Arciniegas, D. B. , Anderson, C. A. , Topkoff, J. , & McAllister, T. W. (2005). Mild traumatic brain injury: A neuropsychiatric approach to diagnosis, evaluation, and treatment. Neuropsychiatric Disease and Treatment, 1, 311–327. 18568112 PMC2424119

[brb3791-bib-0006] Ariza, M. , Pueyo, R. , Matarín Mdel, M. , Junqué, C. , Mataró, M. , Clemente, I. , … Sahuquillo, J. (2006). Influence of APOE polymorphism on cognitive and behavioural outcome in moderate and severe traumatic brain injury. Journal of Neurology, Neurosurgery and Psychiatry, 77, 1191–1193. 10.1136/jnnp.2005.085167 PMC2077553 16614010

[brb3791-bib-0007] Bell, R. D. , Winkler, E. A. , Singh, I. , Sagare, A. P. , Deane, R. , Wu, Z. , … Zlokovic, B. V. (2012). Apolipoprotein E controls cerebrovascular integrity via cyclophilin A. Nature, 485, 512–516. 22622580 10.1038/nature11087 PMC4047116

[brb3791-bib-0008] Berl, M. M. , Balsamo, L. M. , Xu, B. , Moore, E. N. , Weinstein, S. L. , Conry, J. A. , … Gaillard, W. D. (2005). Seizure focus affects regional language networks assessed by fMRI. Neurology, 65, 1604–1611. 16301489 10.1212/01.wnl.0000184502.06647.28

[brb3791-bib-0009] Bigler, E. D. (1987). The clinical significance of cerebral atrophy in traumatic brain injury. Archives of Clinical Neuropsychology, 2, 293–304. 14589621

[brb3791-bib-0010] Bigler, E. D. , Anderson, C. V. , & Blatter, D. D. (2002). Temporal lobe morphology in normal aging and traumatic brain injury. AJNR. American Journal of Neuroradiology, 23, 255–66. 11847051 PMC7975272

[brb3791-bib-0011] Blackman, J. A. , Worley, G. , & Strittmatter, W. J. (2005). Apolipoprotein E and brain injury: Implications for children. Developmental Medicine and Child Neurology, 47, 64–70. 15686292 10.1017/s0012162205000113

[brb3791-bib-0012] Blake, R. V. , Wroe, S. J. , Breen, E. K. , & McCarthy, R. A. (2000). Accelerated forgetting in patients with epilepsy: Evidence for an impairment in memory consolidation. Brain, 123(Pt 3), 472–483. 10686171 10.1093/brain/123.3.472

[brb3791-bib-0013] Blennow, K. , Mattsson, N. , Schöll, M. , Hansson, O. , & Zetterberg, H. (2015). Amyloid biomarkers in Alzheimer's disease. Trends in Pharmacological Sciences, 36, 297–309. 25840462 10.1016/j.tips.2015.03.002

[brb3791-bib-0014] Carroll, L. J. , Cassidy, J. D. , Peloso, P. M. , Borg, J. , von Holst, H. , Holm, L. , … WHO Collaborating Centre Task Force on Mild Traumatic Brain Injury. (2004). Prognosis for mild traumatic brain injury: Results of the WHO Collaborating Centre Task Force on Mild Traumatic Brain Injury. Journal of Rehabilitation Medicine, 43(Suppl), 84–105. 10.1080/16501960410023859 15083873

[brb3791-bib-0015] Cassidy, J. D. , Carroll, L. J. , Peloso, P. M. , Borg, J. , von Holst, H. , Holm, L. , … WHO Collaborating Centre Task Force on Mild Traumatic Brain Injury . (2004). Incidence, risk factors and prevention of mild traumatic brain injury: Results of the WHO Collaborating Centre Task Force on Mild Traumatic Brain Injury. Journal of Rehabilitation Medicine, 43(Suppl), 28–60. 10.1080/16501960410023732 15083870

[brb3791-bib-0016] Chamelian, L. , Reis, M. , & Feinstein, A. (2004). Six‐month recovery from mild to moderate Traumatic Brain Injury: The role of APOE‐epsilon 4 allele. Brain, 127, 2621–2628. 15496436 10.1093/brain/awh296

[brb3791-bib-0017] Chiang, M. F. , Chang, J. G. , & Hu, C. J. (2003). Association between apolipoprotein E genotype and outcome of traumatic brain injury. Acta Neurochirurgica, 145, 649–653, discussion 53‐4. 14520543 10.1007/s00701-003-0069-3

[brb3791-bib-0018] Crawford, F. C. , Vanderploeg, R. D. , Freeman, M. J. , Singh, S. , Waisman, M. , Michaels, L. , … Mullan, M. J. (2002). APOE genotype influences acquisition and recall following traumatic brain injury. Neurology, 58, 1115–1118. 11940706 10.1212/wnl.58.7.1115

[brb3791-bib-0019] Davis, J. J. (2016). Reconsidering the word memory test as a memory measure in traumatic brain injury. Archives of Clinical Neuropsychology, 31, 802–810. 10.1093/arclin/acw058 27538439

[brb3791-bib-0020] Delis, D. C. , Kramer, J. H. , Kaplan, E. , & Ober, B. A. (2000). California verbal learning test, 2nd ed. San Antonio, TX, USA: Psychological Corporation.

[brb3791-bib-0021] Dotson, V. M. , Kitner‐Triolo, M. H. , Evans, M. K. , & Zonderman, A. B. (2009). Effects of race and socioeconomic status on the relative influence of education and literacy on cognitive functioning. Journal of the International Neuropsychological Society, 15, 58–59. 10.1017/S1355617709090821 PMC2722437 19573276

[brb3791-bib-0022] Duhaime, A. C. , Gean, A. D. , Haacke, E. M. , Hicks, R. , Wintermark, M. , Mukherjee, P. , … Common Data Elements Neuroimaging Working Group Members, Pediatric Working Group Members . (2010). Common data elements in radiologic imaging of traumatic brain injury. Archives of Physical Medicine and Rehabilitation, 91, 1661–1666. 21044709 10.1016/j.apmr.2010.07.238

[brb3791-bib-0023] Eramudugolla, R. , Bielak, A. A. , Bunce, D. , Easteal, S. , Cherbuin, N. , & Anstey, K. J. (2014). Long‐term cognitive correlates of traumatic brain injury across adulthood and interactions with APOE genotype, sex, and age cohorts. Journal of the International Neuropsychological Society, 20, 444–454. 24670469 10.1017/S1355617714000174

[brb3791-bib-0024] Fagan, A. M. , & Holtzman, D. M. (2000). Astrocyte lipoproteins, effects of apoE on neuronal function, and role of apoE in amyloid‐beta deposition in vivo. Microscopy Research and Technique, 50, 297–304. 10936884 10.1002/1097-0029(20000815)50:4<297::AID-JEMT9>3.0.CO;2-C

[brb3791-bib-0025] Farrer, L. A. , Cupples, L. A. , Haines, J. L. , Hyman, B. , Kukull, W. A. , Mayeux, R. , … van Duijn, C. M. (1997). Effects of age, sex, and ethnicity on the association between apolipoprotein E genotype and Alzheimer disease. A meta‐analysis. APOE and Alzheimer Disease Meta Analysis Consortium. JAMA, 278, 1349–1356. 9343467

[brb3791-bib-0026] Friedman, G. , Froom, P. , Sazbon, L. , Grinblatt, I. , Shochina, M. , Tsenter, J. , … Groswasser, Z. (1999). Apolipoprotein E‐epsilon4 genotype predicts a poor outcome in survivors of traumatic brain injury. Neurology, 52, 244–248. 9932938 10.1212/wnl.52.2.244

[brb3791-bib-0027] Gasquoine, P. G. (2009). Race‐norming of neuropsychological tests. Neuropsychology Review, 19, 250–262. 19294515 10.1007/s11065-009-9090-5

[brb3791-bib-0028] Gavett, B. E. , John, S. E. , Gurnani, A. S. , Bussell, C. A. , & Saurman, J. L. (2015). The Role of Alzheimer's and Cerebrovascular Pathology in Mediating the Effects of Age, Race, and Apolipoprotein E Genotype on Dementia Severity in Pathologically‐Confirmed Alzheimer's Disease. Journal of Alzheimer's Disease, 49, 531–45. 10.3233/JAD-150252 PMC4858176 26444761

[brb3791-bib-0029] Graham, D. I. , Lawrence, A. E. , Adams, J. H. , Doyle, D. , & McLellan, D. R. (1988). Brain damage in fatal non‐missile head injury without high intracranial pressure. Journal of Clinical Pathology, 41, 34–37. 3343378 10.1136/jcp.41.1.34 PMC1141332

[brb3791-bib-0030] Green, S. B. (1991). How many subjects does it take to do a regression analysis? Multivariate Behavioral Research, 26, 499–510. 26776715 10.1207/s15327906mbr2603_7

[brb3791-bib-0031] Greenberg, S. M. , Rebeck, G. W. , Vonsattel, J. P. , Gomez‐Isla, T. , & Hyman, B. T. (1995). Apolipoprotein E epsilon 4 and cerebral hemorrhage associated with amyloid angiopathy. Annals of Neurology, 38, 254–259. 7654074 10.1002/ana.410380219

[brb3791-bib-0032] Grocott, H. P. , Newman, M. F. , El‐Moalem, H. , Bainbridge, D. , Butler, A. , & Laskowitz, D. T. (2001). Apolipoprotein E genotype differentially influences the proinflammatory and anti‐inflammatory response to cardiopulmonary bypass. Journal of Thoracic and Cardiovascular Surgery, 122, 622–623. 11547323 10.1067/mtc.2001.115152

[brb3791-bib-0033] Gu, Y. , Razlighi, Q. R. , Zahodne, L. B. , Janicki, S. C. , Ichise, M. , Manly, J. J. , … Stern, Y. (2015). Brain Amyloid Deposition and Longitudinal Cognitive Decline in Nondemented Older Subjects: Results from a Multi‐Ethnic Population. PLoS ONE, 10, e0123743. 26221954 10.1371/journal.pone.0123743 PMC4519341

[brb3791-bib-0034] Halliday, M. R. , Rege, S. V. , Ma, Q. , Zhao, Z. , Miller, C. A. , Winkler, E. A. , & Zlokovic, B. V. (2016). Accelerated pericyte degeneration and blood‐brain barrier breakdown in apolipoprotein E4 carriers with Alzheimer's disease. Journal of Cerebral Blood Flow and Metabolism, 36, 216–227. 25757756 10.1038/jcbfm.2015.44 PMC4758554

[brb3791-bib-0035] Han, S. D. , Drake, A. I. , Cessante, L. M. , Jak, A. J. , Houston, W. S. , Delis, D. C. , … Bondi, M. W. (2007). Apolipoprotein E and traumatic brain injury in a military population: Evidence of a neuropsychological compensatory mechanism? Journal of Neurology, Neurosurgery and Psychiatry, 78, 1103–1108. 10.1136/jnnp.2006.108183 PMC2117544 17287237

[brb3791-bib-0036] Hanks, R. A. , Jackson, A. M. , & Crisanti, L. K. (2016). Predictive validity of a brief outpatient neuropsychological battery in individuals 1‐25 years post traumatic brain injury. The Clinical Neuropsychologist, 30, 1074–1086. 27270111 10.1080/13854046.2016.1194479

[brb3791-bib-0037] Hauser, P. S. , & Ryan, R. O. (2013). Impact of Apolipoprotein E on Alzheimer's Disease. Current Alzheimer Research, 10, 809–817. 23919769 10.2174/15672050113109990156 PMC3995977

[brb3791-bib-0038] Howell, D. (2013). Statistical methods for psychology, 8th ed. Belmont, CA, USA: Wadsworth Co.

[brb3791-bib-0039] Ignatius, M. J. , Gebicke‐Härter, P. J. , Skene, J. H. , Schilling, J. W. , Weisgraber, K. H. , Mahley, R. W. , & Shooter, E. M. (1986). Expression of apolipoprotein E during nerve degeneration and regeneration. Proceedings of the National Academy of Sciences of the United States of America, 83, 1125–1129. 2419900 10.1073/pnas.83.4.1125 PMC323024

[brb3791-bib-0040] Isoniemi, H. , Tenovuo, O. , Portin, R. , Himanen, L. , & Kairisto, V. (2006). Outcome of traumatic brain injury after three decades‐relationship to ApoE genotype. Journal of Neurotrauma, 23, 1600–1608. 17115907 10.1089/neu.2006.23.1600

[brb3791-bib-0041] Jacobs, B. , Beems, T. , Stulemeijer, M. , van Vugt, A. B. , van der Vliet, T. M. , Borm, G. F. , & Vos, P. E. (2010). Outcome prediction in mild traumatic brain injury: Age and clinical variables are stronger predictors than CT abnormalities. Journal of Neurotrauma, 27, 655–668. 20035619 10.1089/neu.2009.1059

[brb3791-bib-0042] Jiang, Y. , Sun, X. , Gui, L. , Xia, Y. , Tang, W. , Cao, Y. , & Gu, Y. (2007). Correlation between APOE ‐491AA promoter in epsilon4 carriers and clinical deterioration in early stage of traumatic brain injury. Journal of Neurotrauma, 24, 1802–1810. 18159991 10.1089/neu.2007.0299

[brb3791-bib-0043] Jiang, Y. , Sun, X. , Xia, Y. , Tang, W. , Cao, Y. , & Gu, Y. (2006). Effect of APOE polymorphisms on early responses to traumatic brain injury. Neuroscience Letters, 408, 155–158. 16997460 10.1016/j.neulet.2006.08.082

[brb3791-bib-0044] Kashluba, S. , Hanks, R. A. , Casey, J. E. , & Millis, S. R. (2008). Neuropsychologic and functional outcome after complicated mild traumatic brain injury. Archives of Physical Medicine and Rehabilitation, 89, 904–911. 18452740 10.1016/j.apmr.2007.12.029

[brb3791-bib-0045] Kaup, A. R. , Nettiksimmons, J. , Harris, T. B. , Sink, K. M. , Satterfield, S. , Metti, A. L. , … Aging, Body Composition (Health ABC) Study. (2015). Cognitive resilience to apolipoprotein E ε4: Contributing factors in black and white older adults. JAMA Neurology, 72, 340–348. 25599330 10.1001/jamaneurol.2014.3978 PMC4624320

[brb3791-bib-0046] Kay, A. D. , Day, S. P. , Kerr, M. , Nicoll, J. A. , Packard, C. J. , & Caslake, M. J. (2003). Remodeling of cerebrospinal fluid lipoprotein particles after human traumatic brain injury. Journal of Neurotrauma, 20, 717–723. 12965051 10.1089/089771503767869953

[brb3791-bib-0047] Kern, S. , Mehlig, K. , Kern, J. , Zetterberg, H. , Thelle, D. , Skoog, I. , … Börjesson‐Hanson, A. (2015). The distribution of apolipoprotein E genotype over the adult lifespan and in relation to country of birth. American Journal of Epidemiology, 181, 214–217. 25609095 10.1093/aje/kwu442

[brb3791-bib-0048] Kerr, M. E. , Kraus, M. , Marion, D. , & Kamboh, I. (1999). Evaluation of apolipoprotein E genotypes on cerebral blood flow and metabolism following traumatic brain injury. Advances in Experimental Medicine and Biology, 471, 117–124. 10659138 10.1007/978-1-4615-4717-4_14

[brb3791-bib-0049] Lange, K. L. , Bondi, M. W. , Salmon, D. P. , Galasko, D. , Delis, D. C. , Thomas, R. G. , & Thal, L. J. (2002). Decline in verbal memory during preclinical Alzheimer's disease: Examination of the effect of APOE genotype. Journal of the International Neuropsychological Society, 8, 943–955. 12405546 10.1017/s1355617702870096 PMC1621042

[brb3791-bib-0050] Lenihan, M. W. , & Jordan, B. D. (2015). The clinical presentation of chronic traumatic encephalopathy. Current Neurology and Neuroscience Reports, 15, 23. 25772999 10.1007/s11910-015-0541-5

[brb3791-bib-0051] Liberman, J. N. , Stewart, W. F. , Wesnes, K. , & Troncoso, J. (2002). Apolipoprotein E epsilon 4 and short‐term recovery from predominantly mild brain injury. Neurology, 58, 1038–1044. 11940689 10.1212/wnl.58.7.1038

[brb3791-bib-0052] Lynch, J. R. , Pineda, J. A. , Morgan, D. , Zhang, L. , Warner, D. S. , Benveniste, H. , & Laskowitz, D. T. (2002). Apolipoprotein E affects the central nervous system response to injury and the development of cerebral edema. Annals of Neurology, 51, 113–117. 11782990 10.1002/ana.10098

[brb3791-bib-0053] Maas, A. I. , Harrison‐Felix, C. L. , Menon, D. , Adelson, P. D. , Balkin, T. , Bullock, R. , … Schwab, K. (2010). Common data elements for traumatic brain injury: Recommendations from the interagency working group on demographics and clinical assessment. Archives of Physical Medicine and Rehabilitation, 91, 1641–1649. 21044707 10.1016/j.apmr.2010.07.232

[brb3791-bib-0054] Maas, A. I. , Menon, D. K. , Steyerberg, E. W. , Citerio, G. , Lecky, F. , Manley, G. T. , … CENTER‐TBI Participants and Investigators . (2015). Collaborative European NeuroTrauma Effectiveness Research in Traumatic Brain Injury (CENTER‐TBI): A prospective longitudinal observational study. Neurosurgery, 76, 67–80. 25525693 10.1227/NEU.0000000000000575

[brb3791-bib-0055] Manley, G. T. , Diaz‐Arrastia, R. , Brophy, M. , Engel, D. , Goodman, C. , Gwinn, K. , … Hayes, R. L. (2010). Common data elements for traumatic brain injury: Recommendations from the biospecimens and biomarkers working group. Archives of Physical Medicine and Rehabilitation, 91, 1667–1672. 21044710 10.1016/j.apmr.2010.05.018

[brb3791-bib-0056] Marshall, L. F. , Marshall, S. B. , Klauber, M. R. , Van Berkum Clark, M. , Eisenberg, H. , Jane, J. A. , … Foulkes, M. A. (1992). The diagnosis of head injury requires a classification based on computed axial tomography. Journal of Neurotrauma, 9(Suppl 1), S287–292. 1588618

[brb3791-bib-0057] Merritt, V. C. , & Arnett, P. A. (2016). Apolipoprotein E (APOE) ϵ4 Allele Is Associated with Increased Symptom Reporting Following Sports Concussion. Journal of the International Neuropsychological Society, 22, 89–94. 26483005 10.1017/S1355617715001022

[brb3791-bib-0058] Merritt, V. C. , Rabinowitz, A. R. , & Arnett, P. A. (2017). The influence of the apolipoprotein E (APOE) gene on subacute post‐concussion neurocognitive performance in college athletes. Archives of Clinical Neuropsychology, 24, 1–11. 10.1093/arclin/acx051 28541413

[brb3791-bib-0059] Methia, N. , André, P. , Hafezi‐Moghadam, A. , Economopoulos, M. , Thomas, K. L. , & Wagner, D. D. (2001). ApoE deficiency compromises the blood brain barrier especially after injury. Molecular Medicine, 7, 810–815. 11844869 PMC1950012

[brb3791-bib-0060] Millar, K. , Nicoll, J. A. , Thornhill, S. , Murray, G. D. , & Teasdale, G. M. (2003). Long term neuropsychological outcome after head injury: Relation to APOE genotype. Journal of Neurology, Neurosurgery and Psychiatry, 74, 1047–1052. 10.1136/jnnp.74.8.1047 PMC1738588 12876232

[brb3791-bib-0061] Moran, L. M. , Taylor, H. G. , Ganesalingam, K. , Gastier‐Foster, J. M. , Frick, J. , Bangert, B. , … Yeates, K. O. (2009). Apolipoprotein E4 as a predictor of outcomes in pediatric mild traumatic brain injury. Journal of Neurotrauma, 26, 1489–1495. 19645623 10.1089/neu.2008.0767 PMC2822810

[brb3791-bib-0062] Müller, K. , Ingebrigtsen, T. , Wilsgaard, T. , Wikran, G. , Fagerheim, T. , Romner, B. , & Waterloo, K. (2009). Prediction of time trends in recovery of cognitive function after mild head injury. Neurosurgery, 64, 698–704; discussion 704. 19349827 10.1227/01.NEU.0000340978.42892.78

[brb3791-bib-0063] Nathan, B. P. , Bellosta, S. , Sanan, D. A. , Weisgraber, K. H. , Mahley, R. W. , & Pitas, R. E. (1994). Differential effects of apolipoproteins E3 and E4 on neuronal growth in vitro. Science, 264, 850–852. 8171342 10.1126/science.8171342

[brb3791-bib-0064] Nathoo, N. , Chetry, R. , van Dellen, J. R. , Connolly, C. , & Naidoo, R. (2003). Apolipoprotein E polymorphism and outcome after closed traumatic brain injury: Influence of ethnic and regional differences. Journal of Neurosurgery, 98, 302–306. 12593615 10.3171/jns.2003.98.2.0302

[brb3791-bib-0065] Nicoll, J. A. , Roberts, G. W. , & Graham, D. I. (1995). Apolipoprotein E epsilon 4 allele is associated with deposition of amyloid beta‐protein following head injury. Nature Medicine, 1, 135–137. 10.1038/nm0295-135 7585009

[brb3791-bib-0066] Noe, E. , Ferri, J. , Colomer, C. , Moliner, B. , & Chirivella, J. (2010). APOE genotype and verbal memory recovery during and after emergence from post‐traumatic amnesia. Brain Injury, 24, 886–892. 20377344 10.3109/02699051003724952

[brb3791-bib-0067] Orihara, Y. , & Nakasono, I. (2002). Induction of apolipoprotein E after traumatic brain injury in forensic autopsy cases. International Journal of Legal Medicine, 116, 92–98. 12056527 10.1007/s00414-001-0265-8

[brb3791-bib-0068] Padgett, C. R. , Summers, M. J. , Vickers, J. C. , McCormack, G. H. , & Skilbeck, C. E. (2016). Exploring the effect of the apolipoprotein E (APOE) gene on executive function, working memory, and processing speed during the early recovery period following traumatic brain injury. Journal of Clinical and Experimental Neuropsychology, 38, 551–560. 26898659 10.1080/13803395.2015.1137557

[brb3791-bib-0069] Pan, J. , Connolly, I. D. , Dangelmajer, S. , Kintzing, J. , Ho, A. L. , & Grant, G. (2016). Sports‐related brain injuries: Connecting pathology to diagnosis. Neurosurgical Focus, 40, E14. 10.3171/2016.1.FOCUS15607 27032917

[brb3791-bib-0070] Paolo, A. M. , Troster, A. I. , & Ryan, J. J. (1997). California Verbal Learning Test: Normative data for the elderly. Journal of Clinical and Experimental Neuropsychology, 19, 220–234. 9240482 10.1080/01688639708403853

[brb3791-bib-0071] Ponsford, J. , Draper, K. , & Schönberger, M. (2008). Functional outcome 10 years after traumatic brain injury: Its relationship with demographic, injury severity, and cognitive and emotional status. Journal of the International Neuropsychological Society, 14, 233–242. 18282321 10.1017/S1355617708080272

[brb3791-bib-0072] Pruthi, N. , Chandramouli, B. A. , Kuttappa, T. B. , Rao, S. L. , Subbakrishna, D. K. , Abraham, M. P. , … Shankar, S. K. (2010). Apolipoprotein E polymorphism and outcome after mild to moderate traumatic brain injury: A study of patient population in India. Neurology India, 58, 264–269. 20508347 10.4103/0028-3886.63810

[brb3791-bib-0073] Saito, H. , Dhanasekaran, P. , Baldwin, F. , Weisgraber, K. H. , Phillips, M. C. , & Lund‐Katz, S. (2003). Effects of polymorphism on the lipid interaction of human apolipoprotein E. Journal of Biological Chemistry, 278, 40723–40729. 12917433 10.1074/jbc.M304814200

[brb3791-bib-0074] Schefft, B. K. , Dulay, M. F. , Fargo, J. D. , Szaflarski, J. P. , Yeh, H. S. , & Privitera, M. D. (2008). The use of self‐generation procedures facilitates verbal memory in individuals with seizure disorders. Epilepsy & Behavior, 13, 162–168. 18343201 10.1016/j.yebeh.2008.01.012

[brb3791-bib-0075] Shafi, S. , Marquez de la Plata, C. , Diaz‐Arrastia, R. , Shipman, K. , Carlile, M. , Frankel, H. , … Gentilello, L. M. (2007). Racial Disparities in Long‐Term Functional Outcome After Traumatic Brain Injury. The Journal of Trauma, 63, 1263–1270. 18212648 10.1097/TA.0b013e31815b8f00

[brb3791-bib-0076] Stallings, G. , Boake, C. , & Sherer, M. (1995). Comparison of the California Verbal Learning Test and the Rey Auditory Verbal Learning Test in head‐injured patients. Journal of Clinical and Experimental Neuropsychology, 17, 706–712. 8557811 10.1080/01688639508405160

[brb3791-bib-0077] Strittmatter, W. J. , & Bova Hill, C. (2002). Molecular biology of apolipoprotein E. Current Opinion in Lipidology, 13, 119–123. 11891413 10.1097/00041433-200204000-00002

[brb3791-bib-0078] Stulemeijer, M. , van der Werf, S. P. , Borm, G. F. , & Vos, P. E. (2007). Early prediction of favorable recovery six‐months after mild traumatic brain injury. Journal of Neurology, Neurosurgery and Psychiatry, 79, 936–942. 10.1136/jnnp.2007.131250 17951281

[brb3791-bib-0079] Sundstrom, A. , Marklund, P. , Nilsson, L. G. , Cruts, M. , Adolfsson, R. , Van Broeckhoven, C. , & Nyberg, L. (2004). APOE influences on neuropsychological function after mild head injury: Within‐person comparisons. Neurology, 62, 1963–1966. 15184597 10.1212/01.wnl.0000129268.83927.a8

[brb3791-bib-0080] Sundström, A. , Nilsson, L. G. , Cruts, M. , Adolfsson, R. , Van Broeckhoven, C. , & Nyberg, L. (2007). Fatigue before and after mild traumatic brain injury: Pre‐post‐injury comparisons in relation to Apolipoprotein E. Brain Injury, 21, 1049–1054. 17891567 10.1080/02699050701630367

[brb3791-bib-0081] Suri, S. , Heise, V. , Trachtenberg, A. J. , & Mackaey, C. E. (2013). The forgotten APOE allele: A review of the evidence and suggested mechanisms for the protective effect of APOE ε2. Neuroscience and Biobehavioral Reviews, 37, 2878–2886. 24183852 10.1016/j.neubiorev.2013.10.010

[brb3791-bib-0082] Tayim, F. M. , Flashman, L. A. , Wright, M. J. , Roth, R. M. , & McAllister, T. W. (2016). Recovery of episodic memory subprocesses in mild and complicated mild traumatic brain injury at 1 and 12 months post injury. Journal of Clinical and Experimental Neuropsychology, 38, 1005–1014. 27191799 10.1080/13803395.2016.1182968

[brb3791-bib-0083] Teasdale, G. , & Jennett, B. (1974). Assessment of coma and impaired consciousness. A practical scale. Lancet, 2, 81–84. 4136544 10.1016/s0140-6736(74)91639-0

[brb3791-bib-0084] Teasdale, G. M. , Murray, G. D. , & Nicoll, J. A. (2005). The association between ApoE epsilon4, age and outcome after head injury: A prospective cohort study. Brain, 128(Pt 11), 2556–2561. 16033781 10.1093/brain/awh595

[brb3791-bib-0085] Treble‐Barna, A. , Zang, H. , Zhang, N. , Martin, L. J. , Yeates, K. O. , Taylor, H. G. , … Kurowski, B. G. (2016). Does apolipoprotein e4 status moderate the association of family environment with long‐term child functioning following early moderate to severe traumatic brain injury? A preliminary study. Journal of the International Neuropsychological Society, 22, 859–864. 27480909 10.1017/S1355617716000631 PMC5476473

[brb3791-bib-0086] Van der Naalt, J. (2001). Prediction of outcome in mild to moderate head injury: A review. Journal of Clinical and Experimental Neuropsychology, 23, 837–851. 11910548 10.1076/jcen.23.6.837.1018

[brb3791-bib-0087] Vanderploeg, R. D. , Crowell, T. A. , & Curtiss, G. (2001). Verbal learning and memory deficits in traumatic brain injury: Encoding, consolidation, and retrieval. Journal of Clinical and Experimental Neuropsychology, 23, 185–195. 11309672 10.1076/jcen.23.2.185.1210

[brb3791-bib-0088] Wiens, A. N. , Tindall, A. A. , & Crossen, J. R. (1994). California Verbal Learning Test: A normative study. Clinical Neuropsychologist, 8, 75–90.

[brb3791-bib-0089] Wilde, E. A. , Whiteneck, G. G. , Bogner, J. , Bushnik, T. , Cifu, D. X. , Dikmen, S. , … von Steinbuechel, N. (2010). Recommendations for the use of common outcome measures in traumatic brain injury research. Archives of Physical Medicine and Rehabilitation, 91(1650–1660), e17. 10.1016/j.apmr.2010.06.033 21044708

[brb3791-bib-0090] Woods, S. P. , Delis, D. C. , Scott, J. C. , Kramer, J. H. , & Holdnack, J. A. (2006). The California Verbal Learning Test–second edition: Test‐retest reliability, practice effects, and reliable change indices for the standard and alternate forms. Archives of Clinical Neuropsychology, 21, 413–420. 16843636 10.1016/j.acn.2006.06.002

[brb3791-bib-0091] Yue, J. K. , Vassar, M. J. , Lingsma, H. F. , Cooper, S. R. , Okonkwo, D. O. , Valadka, A. B. , … Investigators, T. R. A. C. K.‐T. B. I. (2013). Transforming research and clinical knowledge in traumatic brain injury pilot: Multicenter implementation of the common data elements for traumatic brain injury. Journal of Neurotrauma, 30, 1831–1844. 23815563 10.1089/neu.2013.2970 PMC3814815

[brb3791-bib-0092] Zlatar, Z. Z. , Bischoff‐Grethe, A. , Hays, C. C. , Liu, T. T. , Meloy, M. J. , Rissman, R. A. , … Wierenga, C. E. (2016). Higher Brain Perfusion May Not Support Memory Functions in Cognitively Normal Carriers of the ApoE ε4 Allele Compared to Non‐Carriers. Frontiers in Aging Neuroscience, 8, 151. 27445794 10.3389/fnagi.2016.00151 PMC4919360

