# Supplementary material for: Apolipoprotein E epsilon 4 (APOE‐ε4) genotype is associated with decreased 6‐month verbal memory performance after mild traumatic brain injury
Source: Brain Behav. 2017 Aug 9;7(9):e00791. doi: 10.1002/brb3.791 (PMC5607554; doi:10.1002/brb3.791)
Supplement: Supplementary file 1 [file BRB3-7-e00791-s001.docx]

**Supporting Information, Table 1.**

**Multivariable regression of six-month verbal memory subscales without *APOE-ε4* status in the model**

| **Variable** | ***B* [95% CI]** | **Sig. (*p*)** |
| --- | --- | --- |
| **Immediate Recall Trials 1-5 *(R^2^ = 0.395)*** |  |  |
| Age (per-year) | -0.31 [-0.42, -0.19] | <0.001 |
| Education (per-year) | 0.92 [0.29, 1.56] | 0.005 |
| Sex (male) | -0.77 [-4.58, 3.04] | 0.688 |
| Race (Caucasian) | 5.60 [1.51, 9.69] | 0.008 |
| PMH Seizures (yes) | -4.41 [-9.81, 0.99] | 0.108 |
| LOC (yes/unknown) | 0.77 [-3.77, 5.31] | 0.737 |
| PTA (yes/unknown) | -5.12 [-9.09, -1.15] | 0.012 |
| CT Pathology (yes) | -8.21 [-12.35, -4.07] | <0.001 |
| **Short-Delay Free Recall *(R^2^ = 0.342)*** |  |  |
| Age (per-year) | -0.09 [-0.13, -0.05] | <0.001 |
| Education (per-year) | 0.25 [0.04, 0.45] | 0.018 |
| Sex (male) | -0.17 [-1.38, 1.04] | 0.782 |
| Race (Caucasian) | 1.45 [0.15, 2.75] | 0.029 |
| PMH Seizures (yes) | -1.42 [-3.14, 0.29] | 0.103 |
| LOC (yes/unknown) | 0.44 [-1.00, 1.88] | 0.547 |
| PTA (yes/unknown) | -0.98 [-2.24, 0.29] | 0.128 |
| CT Pathology (yes) | -2.39 [-3.71, -1.08] | <0.001 |
| **Short-Delay Cued Recall *(R^2^ = 0.357)*** |  |  |
| Age (per-year) | -0.08 [-0.11, -0.05] | <0.001 |
| Education (per-year) | 0.19 [0.03, 0.35] | 0.022 |
| Sex (male) | 0.32 [-0.66, 1.29] | 0.522 |
| Race (Caucasian) | 1.22 [0.18, 2.27] | 0.022 |
| PMH Seizures (yes) | -1.55 [-2.93, -0.18] | 0.028 |
| LOC (yes/unknown) | 0.15 [-1.01, 1.31] | 0.793 |
| PTA (yes/unknown) | -1.02 [-2.04, -0.01] | 0.048 |
| CT Pathology (yes) | -1.75 [-2.81, -0.69] | <0.001 |
| **Long-Delay Free Recall *(R^2^ = 0.393)*** |  |  |
| Age (per-year) | -0.09 [-0.12, -0.05] | <0.001 |
| Education (per-year) | 0.25 [0.07, 0.44] | 0.008 |
| Sex (male) | 0.37 [-0.74, 1.48] | 0.514 |
| Race (Caucasian) | 1.78 [0.59, 2.97] | 0.004 |
| PMH Seizures (yes) | -1.61 [-3.18, 0.04] | 0.044 |
| LOC (yes/unknown) | 0.32 [-1.00, 1.64] | 0.634 |
| PTA (yes/unknown) | -1.26 [-2.42, -0.11] | 0.032 |
| CT Pathology (yes) | -2.49 [-3.59, -1.28] | <0.001 |
| **Long-Delay Cued Recall *(R^2^ = 0.358)*** |  |  |
| Age (per-year) | -0.08 [-0.11, -0.05] | <0.001 |
| Education (per-year) | 0.18 [-0.00, 0.35] | 0.045 |
| Sex (male) | 0.15 [-0.87, 1.18] | 0.766 |
| Race (Caucasian) | 1.24 [0.14, 2.35] | 0.028 |
| PMH Seizures (yes) | -1.12 [-2.58, 0.33] | 0.129 |
| LOC (yes/unknown) | 0.18 [-1.05, 1.40] | 0.774 |
| PTA (yes/unknown) | -0.68 [-1.75, 0.39] | 0.212 |
| CT Pathology (yes) | -2.44 [-3.55, -1.32] | <0.001 |

Tier 1 of the hierarchical multivariable linear regression with the response variables being five verbal memory subscales of the California Verbal Learning Test, Second Edition (CVLT-II). This model includes all demographic and injury history predictors. *APOE-ε4* status is not included in this model. Mean increase or decrease (*B)* and associated 95% confidence intervals [95% CI] are reported for each predictor. *APOE*, apolipoprotein E; CT, computed tomography; LOC, loss of consciousness; PMH, prior medical history; PTA, posttraumatic amnesia
